# Tailored Interfaces in Fiber-Reinforced Elastomers: A Surface Treatment Study on Optimized Load Coupling via the Modified Fiber Bundle Debond Technique

**DOI:** 10.3390/polym13010036

**Published:** 2020-12-24

**Authors:** Julia Beter, Boris Maroh, Bernd Schrittesser, Inge Mühlbacher, Thomas Griesser, Sandra Schlögl, Peter Filipp Fuchs, Gerald Pinter

**Affiliations:** 1 Polymer Competence Center Leoben GmbH, Roseggerstrasse 12, 8700 Leoben, Austria; Boris.Maroh@pccl.at (B.M.); Bernd.Schrittesser@pccl.at (B.S.); Inge.Muehlbacher@pccl.at (I.M.); Sandra.Schloegl@pccl.at (S.S.); PeterFilipp.Fuchs@pccl.at (P.F.F.); 2 Chair of Chemistry of Polymeric Materials, Montanuniversitaet Leoben, Otto-Gloeckel Strasse 2, 8700 Leoben, Austria; Thomas.Griesser@unileoben.ac.at; 3 Department of Polymer Engineering and Science, Montanuniversitaet Leoben, Otto-Gloeckel Strasse 2, 8700 Leoben, Austria; Gerald.Pinter@unileoben.ac.at

**Keywords:** fiber-reinforced elastomers, fiber–matrix interface, surface modification, chemical sizing, fiber bundle pull-out test

## Abstract

The interface between the reinforcement and surrounding matrix in a fibrous composite is decisive and critical for maintaining component performance, durability, and mechanical structure properties for load coupling assessment, especially for highly flexible composite materials. The clear trend towards tailored solutions reveals that an in-depth knowledge on surface treating methods to enhance the fiber–matrix interfacial interaction and adhesion properties for an optimized load transfer needs to be ensured. This research aims to quantify the effect of several surface treatments for glass fibers applied in endless fiber-reinforced elastomers with pronounced high deformations. Due to this, the glass fiber surface is directly modified with selected sizings, using a wet chemical treatment, and characterized according to chemical and mechanical aspects. For this purpose, the interfacial adhesion performance between fibers and the surrounding matrix material is investigated by a modified fiber pull-out device. The results clearly show that an optimized surface treatment improves the interface strength and chemical bonding significantly. The fiber pull-out test confirms that an optimized fiber–matrix interface can be enhanced up to 85% compared to standard surface modifications, which distinctly provides the basis of enhanced performances on the component level. These findings were validated by chemical analysis methods and corresponding optical damage analysis.

## 1. Introduction

Flexible composites combine reinforcing fibers with elastomeric matrix resulting in good mechanical properties and stability but still maintain a flexible structure. Completely new applications for those advanced composite material classes considering supplementary biomimetic approaches [1] could be found, e.g., in the field of medical engineering to generate artificial muscles [2], exoskeletons for rehabilitation [3] or aeroelastic skin-like wings [4,5]. To ensure sufficient mechanical performance for these materials and to avoid unwanted stress concentrations [6,7], efficient load transfer between fibers and the matrix [8] as well as excellent fiber–matrix adhesion are required [9,10]. Thus, in-depth knowledge and quantitative investigations of the interface properties [11,12] are essential, which are resulting from the decisive impact due to targeted surface modifications [10] but also from the characteristics of the combined individual components themselves [13,14]. Test methods to study the fiber–matrix interface have been designed for the single fiber (micro scale), fiber bundle (meso scale) and laminate level (macro scale). The single-fiber- and fiber-bundle-level tests provide data that are well related to the interfacial properties [10,11,12,15,16], whereas the data obtained from the laminate level testing are sensitive, e.g., to fiber directions [17] and other laminate processing-related factors [8,18]. To ensure sufficient performance at component level, which is known to consist of a large number of threads and therefore includes numerous boundary conditions [19], it is important to investigate the material behavior at bundle level as a representative volume unit and to define the global structural behavior accordingly [20,21]. In relation to this, a direct correlation between the micro and meso scale tests with the macroscopic composite parts is not feasible [8,22]. The considered methods to investigate the interface properties for composites are mainly carried out with single fiber or fiber bundle tests like the well-known single-fiber push-out test [23,24], single-fiber pull-out test [25,26] and fiber bundle pull-out (FBPO) test [15,27]. Nevertheless, there is still no consistent design or standardization for the interface characterization, and, therefore, comparability between various test setups is only possible within a limited range. Hence, the essential material data obtained from these tests need to be validated [10,14].

Regarding the different test devices, FBPO testing generally provides the potential to conduct measurements faster, easier and with less complexity to estimate the fiber–matrix adhesion with more realistic failure modes (e.g., statistical fiber distribution or fiber–fiber interaction) than the single fiber tests, which, however, require more sensitive handling [8,14] as well as expensive special test equipment [28,29]. In addition, the FBPO tests are significantly influenced by several factors such as (i) the complex stress distribution in a fiber bundle [21,30], (ii) fiber–fiber interaction [29] with more real failure modes [14,31] or (iii) the presence of statistically distributed filaments inside the fiber bundle [32,33]. Due to the variable cross-section areas inside a fiber bundle, these aspects influence the interlaminar shear strength [19,34], and, therefore, the results can be considered more reliable for performance predictions on laminate lay-ups [19]. Previous research was conducted focusing on the verification of a modified FBPO test device for a more precise interface characterization so that even fiber-reinforced elastomers can be handled adequately due to their distinct flexible performance [35]. Moreover, this study revealed that, besides the right choice of the fiber–matrix combination, the sizing on the fibers has a strong impact on the bearable load coupling and further on the composite performance [15,19]. Apart from the common function of stabilization of the pure fibers due to storage, processing or environmental influences [36], the sizing poses the unique possibility to chemically bond materials with completely different properties and can therefore act as a link to achieve an optimum adhesion at the fiber–matrix interface area [13,37,38,39]. This knowledge provides the opportunity to combine controversial materials. In this context, other researchers already have reported problems with an inadequate fiber–elastomer adhesion and demonstrated that the understanding of fiber sizing and fiber–rubber adhesion is essential to produce high-quality specimens and reliable results for further technical applications [40,41,42]. For example, conventional glass fibers (GF) are typically coated with a silane-based sizing mixture, which is chosen mostly due to economic aspects to generate a widespread usage in industry for several thermoset applications [37]. Subsequently, this treatment obtained a good adhesion, e.g., to polyurethane matrix but compared to that offering a negative effect on the adhesion with silicone rubber, which confirms the use of an appropriate primer [43]. Sufficient wetting of the fibers by the matrix material as well as good adhesion between the surface sizing and polymer-based matrix are required to ensure good mechanical performance in terms of load bearing in composite materials [44,45,46]. Several studies report on the essential influence of the fiber surface treatment, since a tailored adhesive fiber–matrix bonding emphasizes significant differences in the static and dynamic mechanical properties of a composite [47,48]. As such, this strongly influences the force transmission and load coupling between reinforcing structures and their surrounding matrix material [49]. Based on this knowledge, a custom-made surface modification is crucial to achieve an optimized performance of the composite depending on the requirements and material combination. Established surface modification methods for glass fibers include plasma techniques [50] and chemical approaches such as coating or covalent attachment of selected functional coupling agents [51]. In particular, silanization reactions proved to be an ideal method for tailoring surfaces of glass fibers [52]. Previous studies conducted on FBPO tests have already demonstrated that the removal of the fiber treatment that originated from the supplier (more specialized for thermoset application) leads to a distinct improvement in adhesive strength [35]. This research was carried out with the aim of understanding the effect of different sizing and their impact on the fiber–elastomer adhesion as well as on the specific setting of the mechanical properties demonstrated by a fiber pull-out method. In this context, a modified FBPO test setup is presented to enable the characterization of the adhesion behavior in the interface in a fiber-reinforced elastomer. Moreover, the data recording reveals similar measurement sensitivity compared to tests on composite samples, which was proven in previous conducted studies focusing on the influence of fiber orientation and adhesion properties on tailored fiber-reinforced elastomers [34]. Based on this knowledge, it is obvious that pull-out tests on single fibers are indispensable especially for the detailed analysis in the microstructure. Hence, the interface characterization is focused primarily on the main research problem at model scale, and, therefore, unnecessary disruptive factors can be successfully eliminated. However, to predict the interface performance for composite components [48], single fiber tests show an overly high measurement sensitivity, and, thus, experiments on fiber bundles reveal more realistic results [14]. Regarding the advice of a customized fiber surface treatment for optimized fiber matrix adhesion, various silane-based coatings were applied to the same fiber–matrix material combination to demonstrate this impact on the mechanical properties as well as on the further composite performance. Accordingly, different chemical and optical analysis were carried out additionally to prove the modified surface quality. Moreover, an optimized surface modification for flexible composite materials is presented for a tailored load transferability between fiber and elastomeric matrix, which is further important for the load coupling mechanism in flexible composites [19,20].

Thus, the main focus of this study is the feasibility and repeatability of the modified FBPO test to detect the specific bonding properties of fiber–matrix interfaces related to the chemical surface treatments and to provide a qualitative investigation method with an appropriate measurement sensitivity. Therefore, the reliability and proof of the surface modification procedure is determined by the bonding of the immobilized silane groups to the fiber surface and by the covalent reaction with corresponding verification tests, such as zeta potential analysis and X-ray photoelectron spectroscopy (XPS). For a complete survey, a standard industrial surface coating and the pure fiber surface (cleaned and decoated) are analyzed in addition. Subsequently, the interfacial bonding strength, an important parameter, is investigated, where the maximum required force for debonding combined with the pull-out behavior represents the basic performance of the mechanical proprieties. Therefore, the adhesion between the fiber bundles with tailored surface conditions and the elastomeric matrix was investigated with the FBPO test setup as a corresponding verification test. Accompanying optical damage analysis provides a visual confirmation of the failure mode. Overall, this study provides a successful evaluation of reliable material data regarding the fiber–matrix interface performance for subsequent simulations [53] and enables customized data for numerical models on elastic bodies with specific reinforcement structures [19,54]. Furthermore, this can be exploited for several other material clusters based on the requirements of the individual components in the composite. In adaption to this aspect combined with a systematic scaling from micro- to macro-mechanical properties, the profound findings regarding the adhesive fiber–matrix strength resulting in an optimized load coupling in the composite yield the basis for promising approaches, such as tension-twist coupling in fiber-reinforced elastomers [55] for novel smart composite material applications, such as aeroelastic spoilers, flaps or aileron in the aircraft and automotive sectors.

## 2. Materials and Methods

### 2.1. Materials and Chemicals

For the experiments, commercial E-type GF reinforcement provided by CS Interglas AG (Erbach, Germany) with the standardized warp yarn classification EC9-68 × 5 t0 from a single batch and with a 2/2 twill weave was used. The reinforcing structure with an area weight of 220 g/m^2^ ± 5% and an area bundle distribution of 50/50 in the 0°/90° direction was coated with a standard industrial silane-based surface treatment FK144 with a twine thickness of about 68 tex, respectively. The mean diameter of the filament is indicated with approximately 10 µm. Regarding the mechanical properties of the pure reinforcing structure, tensile tests according to the ASTM D2256 [56] were already conducted in detail in previous research focusing on the influence of fiber orientation and adhesion properties of tailored fiber-reinforced elastomers [57]. Pneumatically driven grips with a mandrel shape were considered to ensure good clamping without causing clamp-induced damages among the fixed fiber parts considering a preload of 1 N to ensure identical initial test conditions.

As matrix material, Elastosil RT601 A/B as a polydimethylsiloxane (PDMS) obtained from Wacker Chemie AG (Munich, Germany) was chosen for preparing the bundle pull-out specimens. This PDMS is a vinyl-terminated hyperelastic two-component cast system (the prepolymer, part A and crosslinking system, part B) to analyze the impact on the pull-out behavior due to hyperelasticity (highly flexible elastomers). The polyaddition reaction of PDMS via platinum catalyst involves a hydrosilylation reaction between vinyl-terminated difunctional Si–O groups (part A) and the methylhydrosilane-dimethylsiloxane crosslinker (part B). This hydrosilylation process involves the addition of a silane group to the double bond of the difunctional Si–O group to result in a hyperelastic crosslinked polymer network [58].

Due to the typical inorganic structure as well as organic groups comprising siloxane units, PDMS represents a suitable intermediate position between inorganic and organic compounds. Apart from the high heat, weathering, ozone resistance or good low-temperature flexibility, unfilled PDMS reveals further essential properties due to its low side-chain branching and high free volume within the polymer chains. In this context, by exploiting these structure properties, the higher bonding energy of PDMS in combination with GF can lead to beneficial interface adhesion, which subsequently influences the flexible composite properties, especially for load coupling effects in a specific manner. Hence, these promising findings can be further enhanced by optimized chemical modifications of the fiber surface to investigate the influence and its effects on the fiber–matrix bonding by a tailored fiber surface treatment in more detail. Regarding the technical datasheet, the PDMS with a mixing ration 9:1 (part A: part B) has a density of 1.02 g/cm^3^ and a viscosity of the mixed product (uncured) of 3500 mPas (at room temperature). The pot lifetime was indicated with about 90 min at 23 °C and a hardness of 35 Shore A. Based on the manufacturer’s recommendations, the elastomeric matrix was produced following the respective mixing ratio, including an intermediate pre-degassing vacuum step to avoid air bubbles, followed by a final curing step at 70 °C for 60 min in an air-circulating drying oven. For the experimental study on the mechanical performance of these hyperelastic matrices, tests according to the ISO 37 [59] with type 2 specimens were carried out in previous research [57].

Overall, three different chemical silane-based treatments were considered for tailored GF surface modifications and implemented in the specially developed treatment procedure. The used chemicals for this procedure were anhydrous toluene, anhydrous ethanol and 30 wt.% hydrogen peroxide purchased from VWR International LLC (Radnor, PA, USA), including 96 wt.% sulfuric acid and 30 wt.% ammonium hydroxide solution, which were obtained from Carl Roth GmbH + Co. KG (Karlsruhe, Germany). In terms of the three different silanes, 3-aminopropyltriethoxysilane (APTES), (1H,1H,2H,2H-perfluoro-1-octyl)triethoxysilane (FOTES) and vinyltriethoxysilane (VTES) were provided by Sigma-Aldrich, Inc. (Missouri, MO, USA). All chemicals were applied without any further purifications. For the intermediate washing sequences, deionized water was used over the entire treatment procedure. All of the different fiber surface conditions are listed in Table 1, including a clear labelling also adapted in the further sections.

### 2.2. Desizing Procedure of the Glass Fibers

The treated commercial GF from the supplier contained an organic-based sizing, which was removed by a three-step cleaning method comprising two separate cleaning phases, desizing and activation. In the first step, a part of the coating was removed by placing the extracted GF bundles in the acidic peroxymonosulfuric acid (colloquially also called as piranha solution) consisting of four equivalents of 96 wt.% sulfuric acid (H_2_SO_4_) and one equivalent of 30 wt.% aqueous hydrogen peroxide (H_2_O_2_), in which the fiber bundles were leached and treated for 30 min. After this treatment, the fibers were taken from the acidic piranha solution and repeatedly rinsed with deionized water straight afterwards. In the second step, the cleaned GF were exposed to basic piranha solution comprising one equivalent of 30 wt.% ammonium hydroxide solution (NH_4_OH) and one equivalent of 30 wt.% aqueous H_2_O_2_, where the fibers were treated for 20 min at 60 °C. Once again, the fibers were repeatedly rinsed with deionized water. For the activation, the purified fiber bundles were wetted with anhydrous ethanol (C_2_H_5_OH) and finally dried for 60 min at 120 °C in an air-circulating drying oven. All processing steps were carried out at standard atmosphere conditions according to DIN EN ISO 291 (20 °C, 50% r.h.) [60].

### 2.3. Fiber Surface Modification

For the tailored surface modification step, several cleaned and activated fiber bundles were leached and treated with three different silane-based solutions separately. The solutions consisted either of 1 wt.% solution of FOTES or VTES in anhydrous toluene or 1 wt.% solution of APTES in anhydrous ethanol. Each fiber bundle was treated for 240 min at 60 °C in the respective silane solution followed by the washing step with the corresponding anhydrous solvent (used at the treatment procedure) for three times repeatedly. In the final step, the surface modified fibers were dried for 60 min at 120 °C in an air-circulating drying oven.

### 2.4. Sample Preparation

**Figure 1:**
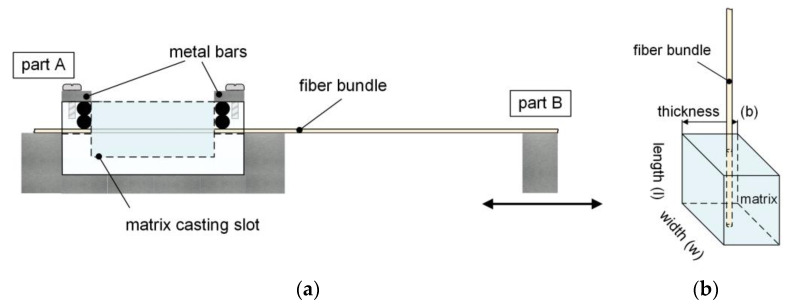
Schematic illustration of the working principle of the manufacturing tool with the main components (**a**) and fiber bundle pull-out (FBPO) specimen (**b**) [35].

In this work, FBPO specimens were prepared for the analysis on tailored fiber–matrix bonding. The modified FBPO test benefits from a fast, easy and economic test condition with a more realistic failure mechanism e.g., statistical filament–matrix distribution, fiber–fiber friction or a more realistic interfacial shear strength distribution. The verification of this presented test setup was described and investigated in detail in another study focusing on a modified FBPO method for the characterization of fiber-reinforced hyperelastic elastomers [35]. For this purpose, a novel specimen manufacturing tool [35] was designed, which had to fulfill the main requirements of (i) an exact fiber bundle positioning in the center of the surrounding matrix material to avoid negative effects caused by tilting or asymmetrical stress distributions along the specimen thickness; (ii) fixing of the fiber bundles, avoiding any pre-damage; (iii) straight placing of the fiber bundle without generating tensile stresses; and (iv) good sealing, especially in the transition region between the embedded and non-embedded fiber bundle due to creep and adhesion forces in fiber bundle direction causing impaired data recording and errors in the results. The handling and assembly design with the main components are schematically illustrated in Figure 1a. Generally, the tool consists of two parts, where part A contains a corpus with narrow slots for the bundles with a defined recess for the cast matrix system. Subsequently, seals have to be implemented on both sides next to the recess to ensure an exact fiber positioning as well as leakage prevention due to the impregnation process. Part B is designed in a movable way with the purpose of aligning the placed fiber bundles in a straight manner. Regarding the surrounding matrix part, all specimens have the exact same geometry with a width *w* of 10 mm, a thickness *b* of 8 mm and a length *l* of 10 mm, where *l* is equal with the embedded length *l*_e_ for the impregnated fiber bundle part (see Figure 1b). Regarding the sample preparation of the FBPO samples, after the casting step of the non-crosslinked prepolymer, the same manufacturing concept was implemented as before, producing the pure elastomeric matrix materials.

### 2.5. Test Setup and Measurement Procedure

**Figure 2:**
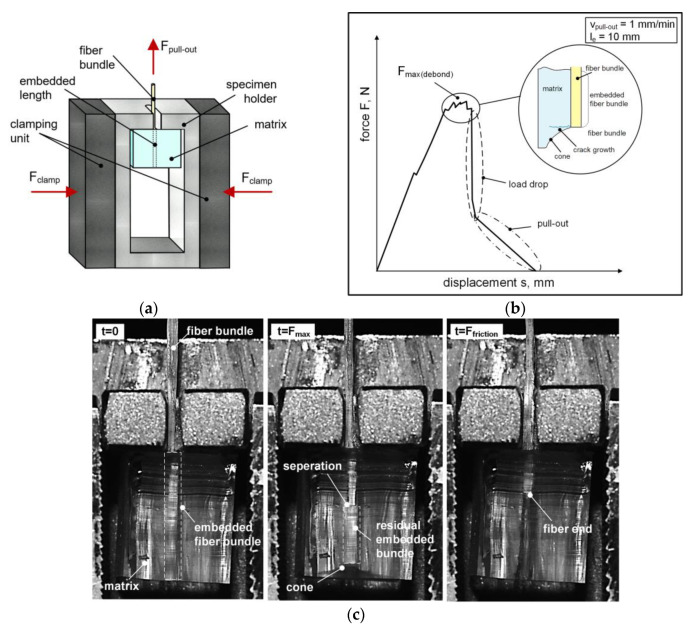
Schematic illustration of the test procedure considering the modified specimen holder (**a**), typical force–displacement graph including the principal performance steps (**b**) and timeline of an FBPO test representing the separation procedure (**c**).

The FBPO tests were performed on a universal testing machine (5500 Series, Instron GmbH, Darmstadt, Germany) using a 100 N load cell, a gauge length of 50 mm and a constant pull-out speed of 1 mm/min. Five reproducible tests per setting within the test plan were conducted to obtain sufficient data for a reasonable statistical evaluation. Moreover, a combination of the ASTM D2256 [56] standard for the fiber bundle (using the same mandrel shaped grips for the pure fiber bundle) with a modified specimen holder (for the surrounding matrix) was implemented, which is depicted in Figure 2a. This holder [35] was specially designed to accommodate the possibility to test with a conventional testing machine (see Figure 2b).

Due to the pronounced flexible behavior of the FBPO specimens with the hyperelastic matrix, further challenges in the test procedure emerged: (i) Clamping of the surrounding matrix has to be avoided to prevent further stresses caused by the grips from resulting in fiber breakage. These stresses would be transmitted through the elastomeric matrix and onward into the embedded fiber bundle. Despite this, (ii) no slippage is permitted to occur, as this could seriously impair the data recording. Since the hyperelastic matrix material is prone to micro surface defects that could cause tilting or twisting, (iii) lateral surfaces have to be considered for the specimen holder. These additional surfaces provide guidance regarding the specimen front surfaces parallel aligned to the inner sides of the modified holder. However, this support is only required at the initial state (until the preload is reached) of each experiment, whilst no contact between the sample and the holder is present, and, thus, sufficient space is available. Hence, the elastic matrix can deform without any additional stresses that could negatively influence the experiment. The FBPO specimens were placed deformation-free inside the specimen holder. To ensure the same testing conditions at initial state and to minimize negative effects e.g., fiber stretch or tilting, a preload of 1 N applied at 1 mm/min was considered. According to the results and the data interpretation, the fiber–matrix adhesion at the interface was determined by recording the load–displacement value, where the maximum occurred load *F*_max_ was set as the significant value for the required pull-out force *F*_max,pull_. For the crucial debonding (indicated by the followed load drop signal), the fiber bundle was loaded until detachment from the surrounding matrix in the interface area emerged, and it was then pulled out completely (followed by the friction-induced pull-out phase).

The FBPO test emphasizes a fiber-loaded configuration resulting in a fiber orientation perpendicular to the matrix surface so that only shear stresses in the fiber–matrix interface is achieved. As discussed in some studies, the elastic deformation energy that is probably released can lead to an initiated step-wise crack growth perpendicular to the longitudinal loading direction (in fiber orientation), which emits during the debonding process [9,12,61] and is schematically explained in Figure 2b. Due to the high elastic behavior especially for PDMS, a cone (see Figure 2c) at the end of the embedded fiber bundle within the sample is clearly observable, whilst the adhesive fiber–matrix bonding to the surrounding matrix is still maintained. Figure 2c shows that the separation mechanism is indicated by the refraction of the light. As expected, the crack initiation begins at the top of the embedded area, whilst only the matrix surface is in contact with the sample holder. 

### 2.6. Surface Characterization

Zeta potential analysis was carried out with the “SurPASS” electrokinetic analyzer (Anton Paar GmbH, Graz, Austria). In general, the streaming potential method was chosen to measure the zeta potential of modified GF in 1mM KCl, whereby the analysis started from the natural pH level to lower acidic values by increasing the titration media up to 50 mM HCl or to higher pH values by increasing it up to 50 mM NaOH with an autotitration unit (RTU, Anton Paar KG, Graz, Austria). The chemical surface analysis of glass fibers was carried out by XPS with a K-Alpha X-ray Photoelectron Spectrometer (Thermo Fisher Scientific Inc., Erlangen, Germany) equipped with a Al-Kα X-ray source (hν = 1486.6 eV). The survey scan was performed with a pass energy of 200 eV and an energy resolution of 1.0 eV. The pass energy amounted to 10 eV (for narrow resolution spectra) with an energy step size of 0.1. The peaks were fitted according to the Gaussian–Lorentzian mixed function considering a Shirley background correction with the provided software of the supplier (Data Analysis Software—Thermo Avantage v5.906, Thermo Scientific, Vienna, Austria).

### 2.7. Optical Damage Analysis

Supplementary optical damage analysis was carried out via light microscope (Axioscope 7, Carl Zeiss GmbH, Graz, Austria) to support the comparability and interpretation of the performed FBPO tests. Due to the high elasticity of the elastomeric matrix material and the above-mentioned crack growth process, a camera system (Prosilica GT 6600, Allied Vision Technologies GmbH, Stadtroda, Germany) was additionally employed for all FPBO tests to improve the correlation of the recorded data of the material behavior during the pull-out with the optical-supported debonding failure process. Subsequently, the following calculation and interpretation of the results were conducted more accurately, which enabled a reliable performance prediction of the adhesive fiber–matrix bonding influenced by different interface modifications.

## 3. Results and Discussion

In previous work, promising and reliable results were achieved using the modified FBPO test setup method for the investigation of different fiber–matrix material combinations [35]. Based on these findings, the emphasis in the following study is on the measurement sensitivity of the modified FBPO test setup towards surface sizings. Therefore, fiber surfaces with varying chemical surface composition were created whilst a constant fiber (GF) matrix (PDMS) combination was applied. With this approach, the influence of controlled surface modifications on the pull-out behavior could be studied in detail. To achieve a tailored adhesion between the glass fibers and the surrounding elastomeric matrix, functional organo-silanes comprising vinyl-, amino- or perfluoro-groups were attached to the fiber surface. Modified GF were obtained with varying surface polarity and chemical functionality, which are expected to distinctively affect the bond strength at the fiber–matrix interface. The applicability of the FBPO test setup was assessed to determine the adhesion strength as a function of the attached silane and to gain a deeper insight into the mechanical properties of fiber–matrix interfaces.

### 3.1. Surface Characterization

**Figure 3:**
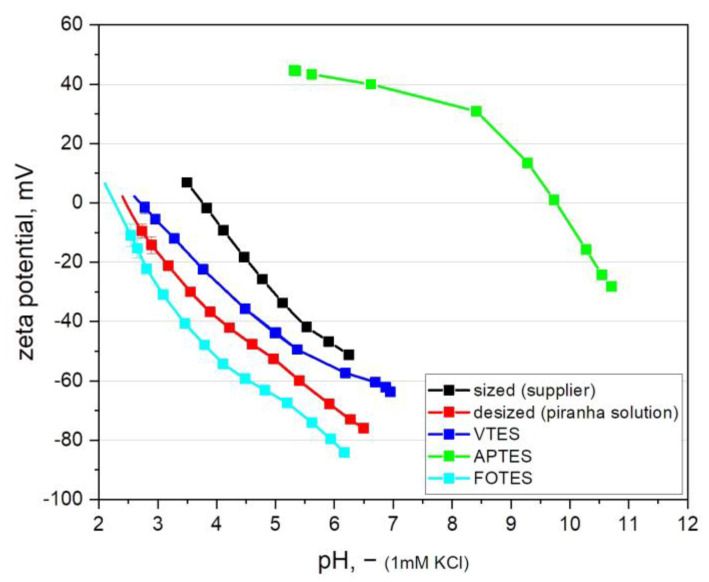
Zeta potential as a function of the pH value of glass fibers prior and after desizing (acidic and basic piranha treatment) and subsequent modification with functional organo-silanes.

Zeta potential measurements were carried out to investigate the change in the surface charges of GF prior to and after the modification procedure (see Figure 3). Commercially available fibers were used with a proprietary sizing, which was removed by a treatment with acidic and basic piranha solution. The results reveal an isoelectric point (IEP) of the GF with the treatment from supplier (prior the desizing step) of about 3.8, indicating the presence of weak basic groups [62]. After the desizing and activation of GF with acidic and basic piranha solution, the IEP shifts to a lower value of about 2.4 [37]. This can be explained by the presence of a high number of acidic silanol moieties of the inorganic GF, which become dominant after the successful removal of the organic sizing during the acidic and basic piranha treatment. Along with the removal of the sizing, the silanol groups were activated on the surface for the subsequent immobilization of the organo-silanes [63]. Figure 3 shows negative zeta potential values, which are characteristic for GF surfaces, since the acidic groups on the surface are fully separated in the basic pH range, resulting in negative potential surface charges [64,65].

Regarding the zeta potential of the modified fibers, the results reveal that the attachment of the perfluorinated silane (FOTES) slightly shifts the IEP to a lower pH value of about 2.2 compared to the IEP of 2.4 of the desized fibers [66]. This result indicates the change in the surface chemistry of GF since FOTES shifts the IEP values towards the acidic region and can lead to a superhydrophobic surface with a significantly low surface energy [67,68]. For the surface modification with the vinyl-functional silane (VTES) a higher IEP of about 2.8 is observed, which is explained by the conversion of acidic silanol groups and the attachment of non-charged and neutral vinyl groups [69]. In contrast to that, the immobilization of organo-silanes with basic amino groups (APTES) significantly increases the pH value of the surface with an indicated IEP of about 9.9 [70]. At low pH values, the amino groups become protonated, whilst an increase in pH causes deprotonation and adsorption of OH^−^ ions, which leads to a negative surface charge in the high pH range [64]. Generally, for all zeta potential results, it should be noted that specimens from fibers possess a higher measurement sensitivity due to their larger surfaces compared to planar samples [71]. Consequently, the small difference between the zeta potential measurements of FOTES, VTES modified fibers and desized fibers in particular was considered as significant.

Moreover, the changes in the chemical surface composition of the modified glass fibers were evidenced by XPS analysis. The detected elements are summarized in Table 2, where all results are referred to the surface composition with the unit in atom-%.

**Figure 4:**
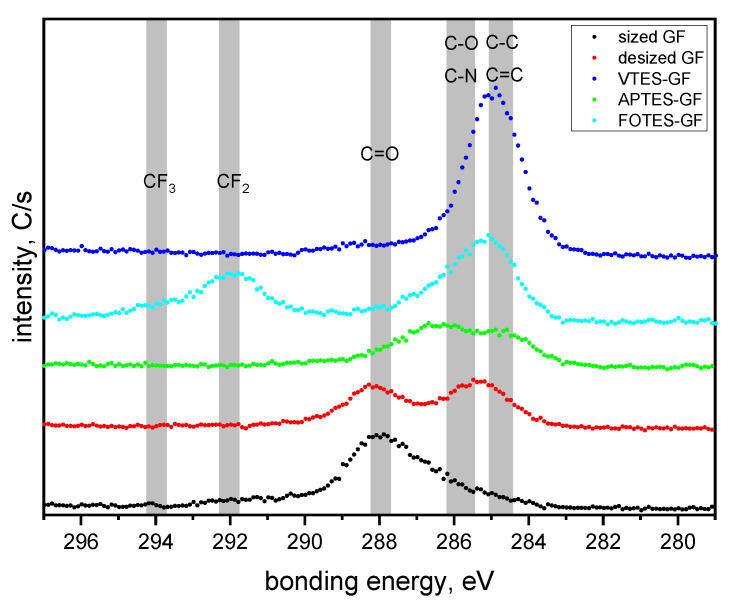
High resolution C 1s spectra of the glass fiber surface prior to and after desizing (acidic and basic piranha treatment) and subsequent modification with functional organo-silanes.

On the surface of the desized GF, C signals corresponding to various carbon species (e.g., C–C, C–O and C=O) are still detectable in the XPS spectrum (Figure 4). The results suggest that the desizing process was not able to fully remove the organic sizing. However, the desizing of the GF leads to a significant increase in the Si and O content compared to the commercially available sized GF, and the formed silanol groups can be exploited as reactive anchor groups for the subsequent coupling of functional organo-silanes. In particular, the attachment of APTES is confirmed by the appearance of the N signal at about 401.6 eV [72], whilst the coupling of FOTES is related to the appearance of the F signal in the characteristic bonding energy region at about 689.7 eV [73]. The comparison of the high resolution C 1s spectra for GF with different surface modifications is given in Figure 4.

The GF sized by the supplier shows two components of the C 1s spectrum related to C–O at 286.2 eV and C=O at 288.0 eV. After the acidic and basic piranha treatment, both characteristic signals for CO_2_ impurities could be observed at 285.5 and 288.1 eV (C–O to C=O ratio of 1 to 0.9). The results confirm that the carbon signal detected in the desized GF is indeed from physisorbed CO_2_ and not related to residues of the organic sizing. The attachment of VTES is associated with an increase in the carbon content on the surface as reported in Table 2, which can be explained by the C–C and C=C bonds (285.0 eV) present in the structure of the organo-silane. In contrast, the APTES-modified GF comprise some CO_2_ impurities and additional signals for C–C bond at 284.6 eV and C–N bond, which was overlapping with the signal for the C–O bond at 286.4 eV [72,74]. For the FOTES modified GF, the results revealed two characteristic signals for the CF_2_ group at 292.0 eV and the CF_3_ group at 294.1 eV [73].

### 3.2. Characterization of Fiber–Matrix Interaction

**Figure 5:**
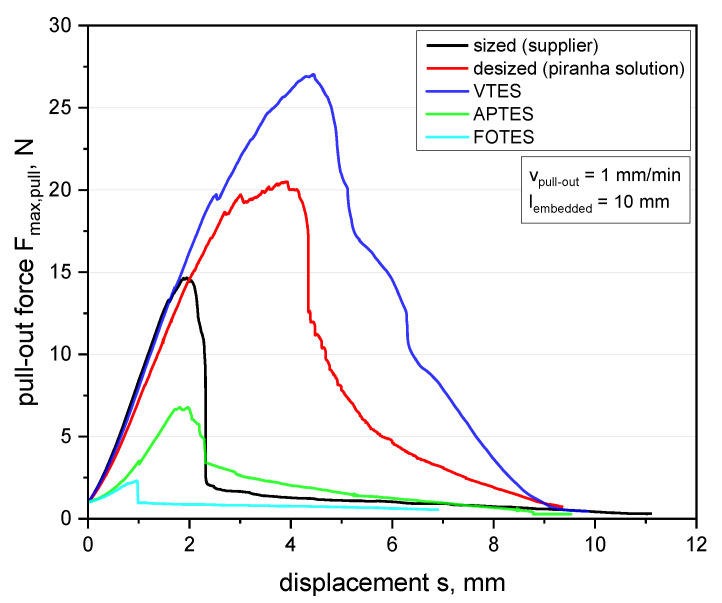
Force–displacement curve determined from the FBPO test on glass fiber (GF) bundles with the polydimethylsiloxane (PDMS) matrix for different fiber surface modifications.

Concerning the pull-out behavior of modified fiber–matrix interfaces versus the maximum bearable load *F*_max,pull_, the results of GF incorporated within a PDMS matrix are graphically compared in Figure 5. As expected, the attached functional groups of FOTES gave the lowest adhesion strength with a maximum force of about 2.5 N due to the hydrophobic nature of perfluorinated surfaces and absence of any chemical interactions (e.g., covalent bonds, H-bonds or ionic interactions). Thus, fiber–matrix interactions were successfully impaired and further physical or other chemical bonding reactions were considerably hindered [67]. The treatment with APTES reveals a clearly lower maximum pull-out forces of about 6.7 N compared to the commercially sized fibers from the supplier with a *F*_max,pull_ of about 14.5 N. This can be explained by the weaker bonding energy of amino groups with the PDMS matrix, and, therefore, this leads to less compatibility, especially in the fiber–matrix interaction [70], which can be clearly observed in Figure 5. However, it can be seen that the removal of the commercial sizing with piranha solution improves the fiber–matrix interface performance significantly, and the maximum pull-out force *F*_max,pull_ amounts to 20.5 N (approximately 40%) compared to commercial sized fibers. We assume that this improved adhesion performance is related to the presence of ketone groups from the oxidized sizing residues, which are known to undergo catalyzed hydrosilylation with Si-H bonds [75]. Thus, a direct coupling of the oxidized residues of the sizing with the PDMS matrix (which is a two-component system containing activated Si–H) is obtained, leading to an enhanced pull-out force. In relation to this, the results for VTES-treated GF reveal the highest adhesion strength between the fiber bundles and the surrounding matrix, indicated by the high required maximum pull-out force of about 27.1 N (approximately 85%) compared to commercially available sized fibers from the supplier. During the hydrosilylation process of the PDMS with the vinyl-terminated silanes, the modified glass fiber surface via VTES additionally reacts with the methylhydrosilane groups of the crosslinker, which leads to good covalent bonding between the GF and the PDMS matrix [58]. This effect can be enhanced by using higher processing temperatures during the sample manufacturing process of about 70 °C, where the bonding of the vinyl groups during the curing steps via platinum catalyst co-reacts and can be proceed more easily. It is evident that results of the FBPO test with the commercial sizing from the supplier reveal an intermediate position compared with all other treated GF-PDMS samples. A reason for this is that those sizings are usually a mixture of various chemicals typically for a broader range of composite application with emphasis on different specifications, such as the economical aspect for large production units, mostly thermoset-based products and medium adhesion to different resin systems [37]. In general, a direct correlation between the tailored silane-treated fibers and the fibers with a commercial sizing should be considered carefully, since the commercial fibers may also contain film-building agents and other components that are responsible for a homogeneous wetting of the fiber surface. Therefore, additional mixed interactions occur, since the influence of chemical interactions due to covalent bonds as well as physical effects, such as polar or non-polar effects or adhesive interactions, are involved.

As an overview, the results of the FBPO tests versus influence of different surface-treated GF are summarized in Table 3 to examine the measurement sensitivity and corresponding pull-out behavior related to the maximum bearable force *F*_max,pull_.

In this context, the FBPO test setup proved to be concise, and significant results with an indicated reasonable standard deviation were obtained. Thus, a reliable data interpretation can be carried out, which is listed in Table 3. Despite various influencing factors, such as the statistical fiber distribution inside the bundle or occurring fiber–fiber friction during the pull-out process, a more realistic failure behavior, especially with an accompanying preliminary analysis of suitable surface modifications depending on the application, can be achieved. Furthermore, it is proven that even with a lower interface adhesion, e.g., with FOTES or APTES modifications, a clear difference in pull-out performance can be observed, which confirms the valid assessment of the FBPO test setup.

**Figure 6:**
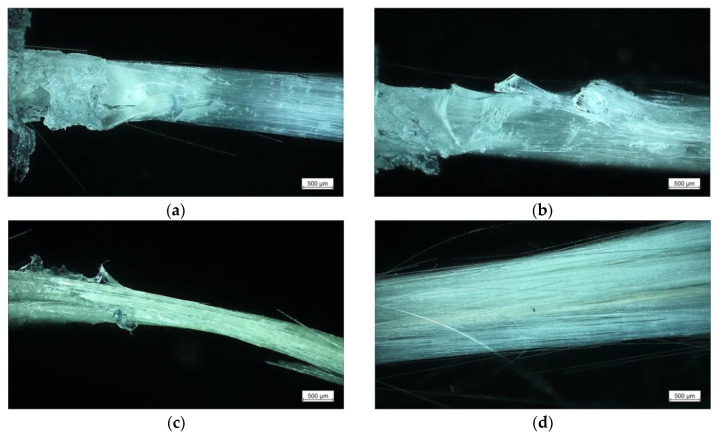
Light micrographs after the fiber bundle pull-out test of VTES-modified GF (**a**), GF desized with acidic and basic piranha solution (**b**), APTES-modified GF (**c**), GF sized from the supplier (**d**).

Besides the results obtained from the FBPO test, the accompanying optical damage analysis provides further information about the pull-out performance and fracture surfaces, which show good agreement with both analysis methods, the zeta potential and FBPO tests. As illustrated in Figure 6a, the optimized interface adhesion of VTES-modified fibers corresponds to the resulting damage surface. It is evident that the complete GF bundle is encapsulated with PDMS, which indicates that the adhesive fiber–matrix bonding is higher than the strength of the matrix. The resulting fracture surface is located near the interface inside the pure PDMS, where the stress concentration of the already deformed elastomer is maximized. Moreover, this effect can be further obtained for desized GF, revealing an adequate fiber–matrix adhesion due to the formed covalent bonds between GF and siloxane monomers of PDMS, which is visible in Figure 6b. In contrast to this, PDMS residues can be barely depicted on the fracture surface of APTES modified GF. This can be explained by the high surface polarity of the amino groups compared to hydrophobic PDMS matrix leading to lower chemical interactions between the treated GF with PDMS (see Figure 6c). Figure 6d shows an example of an original GF bundle (before PDMS wetting) and with the corresponding surface sizing from the supplier.

## 4. Conclusions

In this study, the surface of glass fibers was modified by immobilizing selected organo-silanes to investigate the influence of the chemical surface composition on the fiber–matrix interface and further on the load coupling. Since the research interest in “smart materials” is permanently growing, elastomeric matrix materials with high flexibility and reinforced with stiff glass fibers (GF), in particular with silicone as matrix material, were studied exclusively in this work. Moreover, the emphasis was placed on the assessment and validation of the measurement sensitivity of the modified fiber bundle pull-out device induced by different fiber surface conditions. Three organo-silanes were specifically chosen to create different fiber surface energies, whilst the fiber–matrix material combination was kept constant for all experiments. The modification and desizing of GF were confirmed by zeta potential tests, indicating a clear shift of the corresponding isoelectric point (IEP) from 3.8 to 2.4 at the desized state in the first step and an IEP of about 9.9, 2.2 or 2.8 with the specific surface modifications in the following step. XPS measurements confirmed the changes of the chemical surface composition by the appearance of characteristic signals for N and F and the associated changes in the C 1s spectra. Through zeta potential and XPS measurements, the FBPO test and optical damage analysis, the results reveal that the fiber–matrix interface performance was significantly improved using a vinylsilane-based surface modification, since the maximum bearable load was enhanced from about 14.5 to 27.1 N (85% higher values) compared to the commercially treated fibers from the supplier. This study on FBPO tests achieved that for the same fiber–elastomer material combination including different surface coatings, significant changes in the adhesion and fiber matrix bonding were observed. In this context, the modified FBPO test proved a clear intended difference between the results of the fluoro- and aminosilane-based modifications, which emphasizes the verification of measurement sensitivity and sufficient reproducibility. The attached functionalities were additionally compared with optical damage analysis, which correlates with the results of the pull-out performance, and they were in good agreement with the corresponding mechanical behavior caused by the tailored surface treatments. Thus, an optimized interface adhesion of vinylsilane-modified fibers corresponds to the resulting damage surface, demonstrating an unaffected fiber–matrix interface during debonding and failure Overall, the results demonstrated that the fiber–matrix adhesion was adequately achieved and controlled by a suitable surface modification of the glass fibers. Therefore, besides the choice of single material components (matrix or reinforcing material), an optimized fiber–matrix interface significantly contributes to the load coupling between fibers and the surrounding matrix and further in the performance of composite applications. Based on this research, the important findings concerning tailored fiber surfaces for optimized fiber–matrix interfaces is a crucial part in ongoing studies focusing on load coupling mechanisms triggered in flexible composites. Further studies are in progress to investigate the dependence of optimized interfacial adhesion properties and their impact on the cyclic behavior related to the structure–property interactions. Moreover, these findings provide more precise material parameters which are implemented in accompanying ongoing simulation models for fiber-reinforced elastomers to generate accurate material behavior of composites with distinct flexibility.

## Figures and Tables

**Table 1:** Different surface modifications of glass fibers prior to and after desizing, and attached organo-silane treatments with respective labels.

Surface Modification	Feature	Label
1	commercial sizing FK144	sized
2	piranha treatment	desized
3	vinyltriethoxysilane	VTES
4	3-aminopropyltriethoxysilane	APTES
5	1H,1H,2H,2H-perfluoro-1-octyl)triethoxysilane	FOTES

**Table 2:** Chemical surface composition of glass fibers prior to and after desizing and attachment of functional organo-silanes in atom-%.

	Sized	Desized	VTES	APTES	FOTES
Si	9.4	20.3	11.9	21.4	14.2
C	58.0	33.2	61.8	40.2	25.4
O	32.6	46.5	26.3	35.2	32.3
F	-	-	-	-	28.1
N	-	-	-	3.2	-

**Table 3:** Maximum pull-out force *F*_max,pull_ from FBPO tests for different surface-treated GF bundles combined with the PDMS matrix.

Surface Treatment	Sized (Supplier)	Desized (Piranha Solution)	VTES	APTES	FOTES
max. pull-out force *F*_max,pull_, N	14.5 ± 1.8	20.5 ± 2.4	27.1 ± 2.9	6.7 ± 1.1	2.5 ± 0.5
